# Functionalization of cotton fabric using bio-organic heat storage materials for human protection and thermal comfortability

**DOI:** 10.1038/s41598-025-01328-0

**Published:** 2025-05-22

**Authors:** Menna Zayed, Heba Ghazal, Hanan A. Othman, Eman Abd El-Aziz, Ahmed G. Hassabo

**Affiliations:** 1 Textile Printing, Dyeing and Finishing Department, Faculty of Applied Arts, Benha University, Benha, Egypt; 2 Textile Research and Technology Institute, Pretreatment, and Finishing of Cellulose-Based Fibres Department, National Research Centre (Scopus Affiliation ID 60014618), 33 El-Behouth St. (Former El-Tahrir Str.), P.O. 12622, Dokki, Giza Egypt

**Keywords:** Cotton fabric, Bio-organic heat storage materials, Human protection, Thermal comfortability, Health care, Materials science

## Abstract

In this work, the synthesis of smart hosting materials from modified gelatin with different fatty acids (myristic acid, palmitic acid, and stearic acid) has been observed and characterized. Coconut oil was used as a natural phase change material (PCM) and octadecanol was used as an organic phase change material. Different concentrations of Phase change materials were impregnated in modified gelatin and applied to cotton and/or dyed cotton fabric with reactive dye. The treatment process was done using two methods, first method, the fabric was pre-treated with PCM composite and then dyed with reactive dye solution, and second method, the fabric was dyed with reactive dye solution and then treated with PCM composite. Smart PCM composite based on modified gelatin produced thermo-regulating properties which are responsible for controlling body temperature. The gelatin/PCM composite and treated cotton fabric were characterized using DSC, FT-IR, and SEM. The results confirmed the synthesis of modified gelatin and also confirmed its reaction with the cotton surface. DSC results showed that the treated cotton fabric with coconut oil composite has the best thermo-regulating properties.

## Introduction

Smart fabrics have grown in popularity during the previous decade. New materials have been produced to attain high performance characteristics. Phase change materials (PCMs) are novel substances that are incorporated into textile structures to obtain a thermoregulated fabrics. PCMs are thermal energy storage (TES) materials that can store and release large amounts of latent heat during phase change. There are many kinds of PCMs have been synthesized, including organic and inorganic^1^ and investigated for a variety of applications such as buildings^2^, thermal protection^3^, textiles^4–6^, etc.^7–9^.

The most common problems with PCM materials during use are melting and leaking. Therefore, a hosting material has been developed for their benefit in order to retain the PCMs within its network. There are many different types of hosting materials, some of them have very long side chains that keep PCMs between them and enabling them to perform their functions without leaking. These hosting materials can be chemically or physically attached to the surface. Thus, chemically bonded hosting materials are more preferable since they make the surface permanently treated^10,11^.

PCMs used in textiles are typically microencapsulated into a polymer to avoid material leakage in a liquid condition^12^. Microencapsulation might take place through physical coating methods or chemical methods. Although urea formaldehyde, styrene, polyurethane, melamine formaldehyde, methacrylate, and its copolymers are commonly employed as shell materials in PCM microencapsulation, they could lead to health and environmental issues^13,14^. To tackle this problem, many studies have investigated natural polymers as alternative shell to retain the PCMs within their network.

**Fig. 1:**
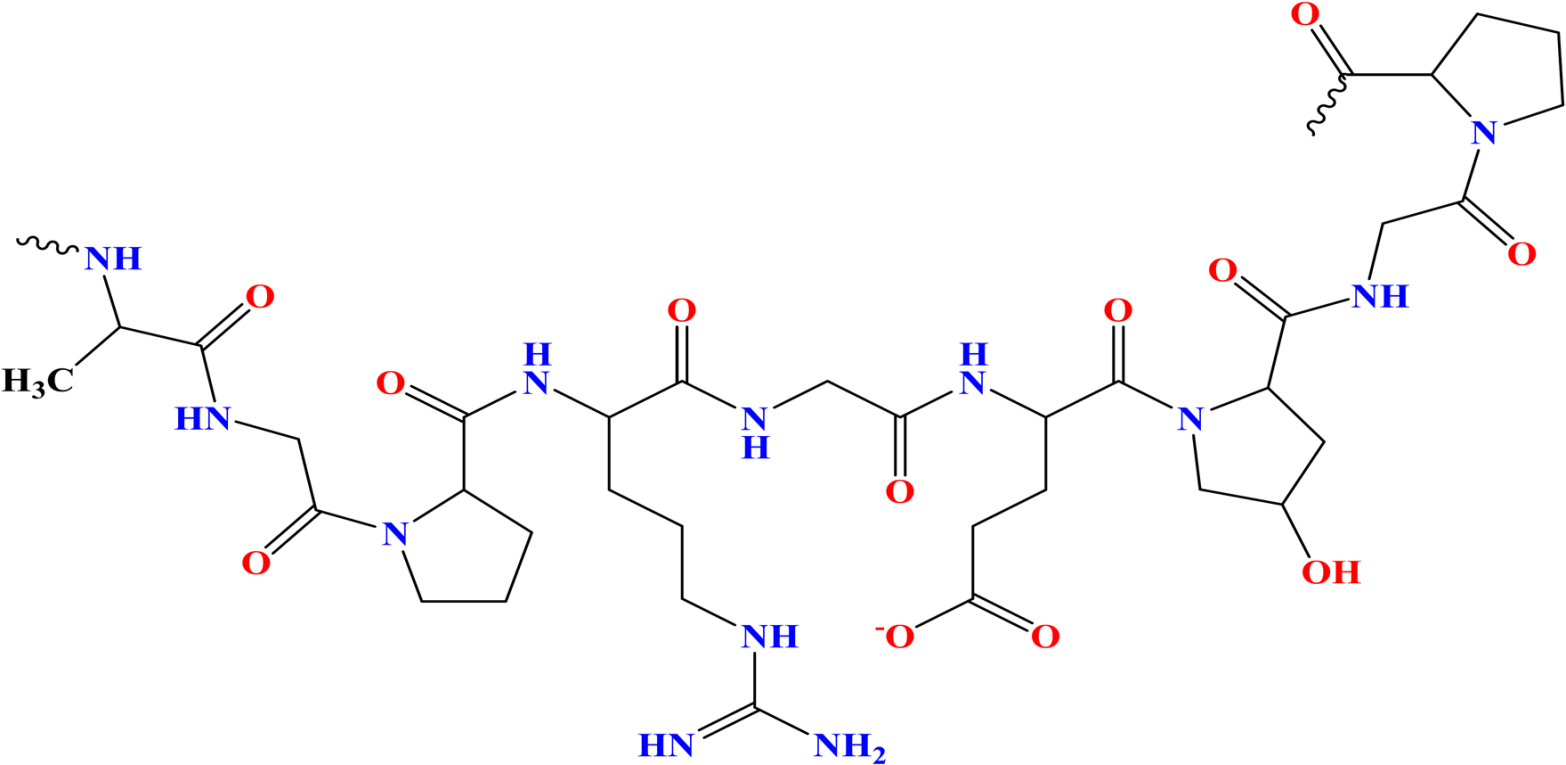
Chemical structure of gelatin.

One of the important natural polymers is gelatin. Gelatin is a polypeptide with a high molecular weight which is non-toxic and biodegradable^15,16^. Gelatin is a soluble protein compound formed when collagen is partially hydrolyzed^17^. Gelatin is made up of eighteen different amino acids, the three most prevalent amino acids in gelatin are proline, hydroxyproline, and glycine. These amino acids form polypeptide chains when they combine. There are more than a thousand amino acids in these polypeptide chains (Fig. 1)^18,19^.

Linear long chain hydrocarbon paraffin waxes, polyethylene glycols, fatty acids, and their mixtures are PCMs that can be used in textiles^12,20–22^. Coconut oil has a high latent heat capacity for TES and it has received little attention in the studies, particularly for textile applications. Coconut oil is a natural source that is derived commercially from copra, which is the flesh of a coconut. From coconut milk, virgin coconut oil is extracted. Coconut oil contains a significant concentration of low molecular weight saturated fatty acids, mostly from lauric oil. Virgin coconut oil includes 46–48% lauric acid and the rest is made up of myristic acid, stearic acid, caproic acid, capric acid, linoleic acid, and caprylic acid^23^. These fatty acids that was found in coconut oil have some merits, such as congruent melting, high latent heat storage capacity, suitable phase change temperature range, favorable thermal and chemical stability, non-toxic and non-flammability^1^.

The goal of this study was to investigate the usage of organic coconut oil as a natural PCM for textile applications as a low-cost, sustainable alternative to presently utilized PCMs like paraffin waxes by contrasting them with those of recognized PCMs (octadecanol) and the usage of gelatin as a bio-hosting material to keep the PCMs inside its chains. Then, further application onto cotton fabric to have thermo-regulated fabric.

## Experimental

### Materials

**Fig. 2:**
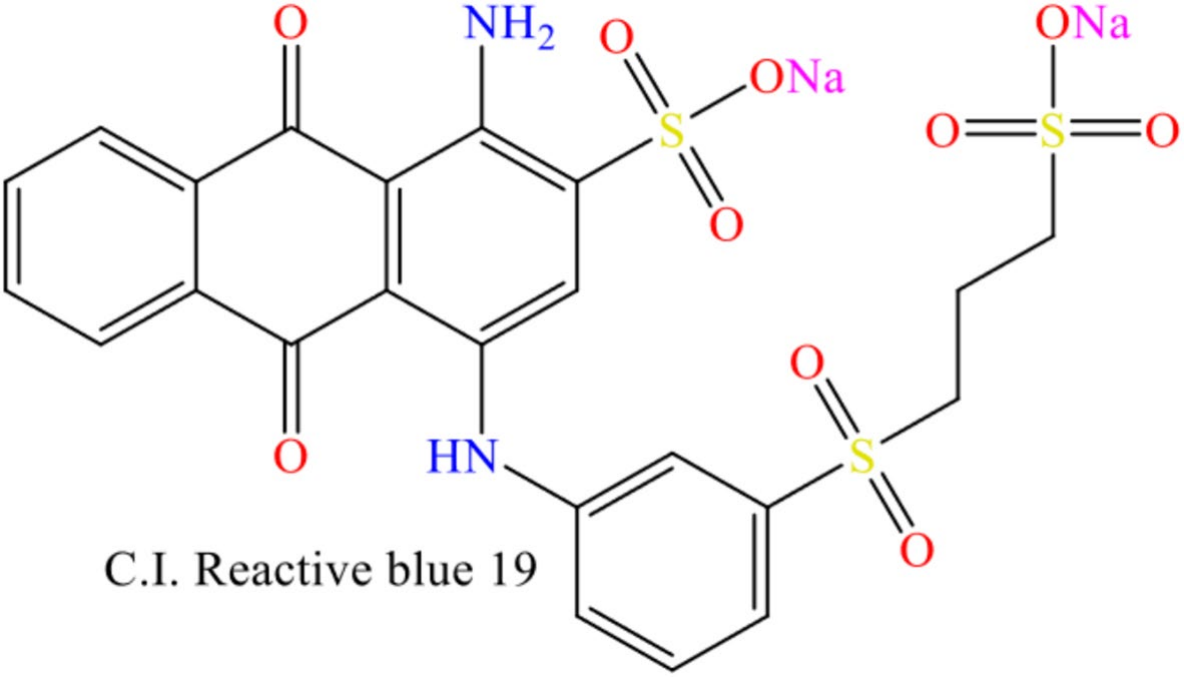
Chemical structure of reactive dye.

Cotton fabric (100%, 265 gm/m^2^) supplied by Al-Qasas Company. stearic palmitic, and myristic acid was provided from Alpha Chemika. coconut oil purchased from Qus natural oil. octadecanol provided from Sigma Aldrich Co., C.I. Reactive blue 19 as a reactive dye were kindly supplied by Dystar Co., Egypt (chemical structure of dye was illustrated in Fig. 2). Gelatin, sodium chloride, tween 80 and sodium hydroxide were purchased from El Nasr pharmaceutical chemicals Co. Citric acid, sodium hypophosphite (SHP) was provided from Fluka. All the reagents and chemicals were used without any purification.

### Methods

#### Synthesis of gelatin/fatty acid ester

Gelatin/fatty acid ester was prepared according to the modified method reported before by Hassabo et al.^10,24,25^ In brief, 10 g gelatin was dissolved in 100 ml of distilled water at 70 °C. Then fatty acids was added to gelatin solution under continuous stirring for 60 min at 160 °C. after that, the mixture was left to cool at room temperature. The final product was dialyzed in deionized water for 1 day and finally lyophilized for 2 days. To adjust the best condition of synthesizing, different fatty acids (stearic, myristic and palmitic acid) was used and added to gelatin with different molar ratios.

#### Synthesis of PCM composite based on gelatin/fatty acid ester

PCM composites were prepared by mixing gelatin fatty acid ester with different PCM materials (coconut oil or octadecanol) in different molar ratios (10, 20 and 30% (w/w) PCM material: polymer) at 90 °C for 2 h, then PCM composite was employed for further investigation and application.

#### Treatment of cotton fabric with PCM composite

**Fig. 3:**
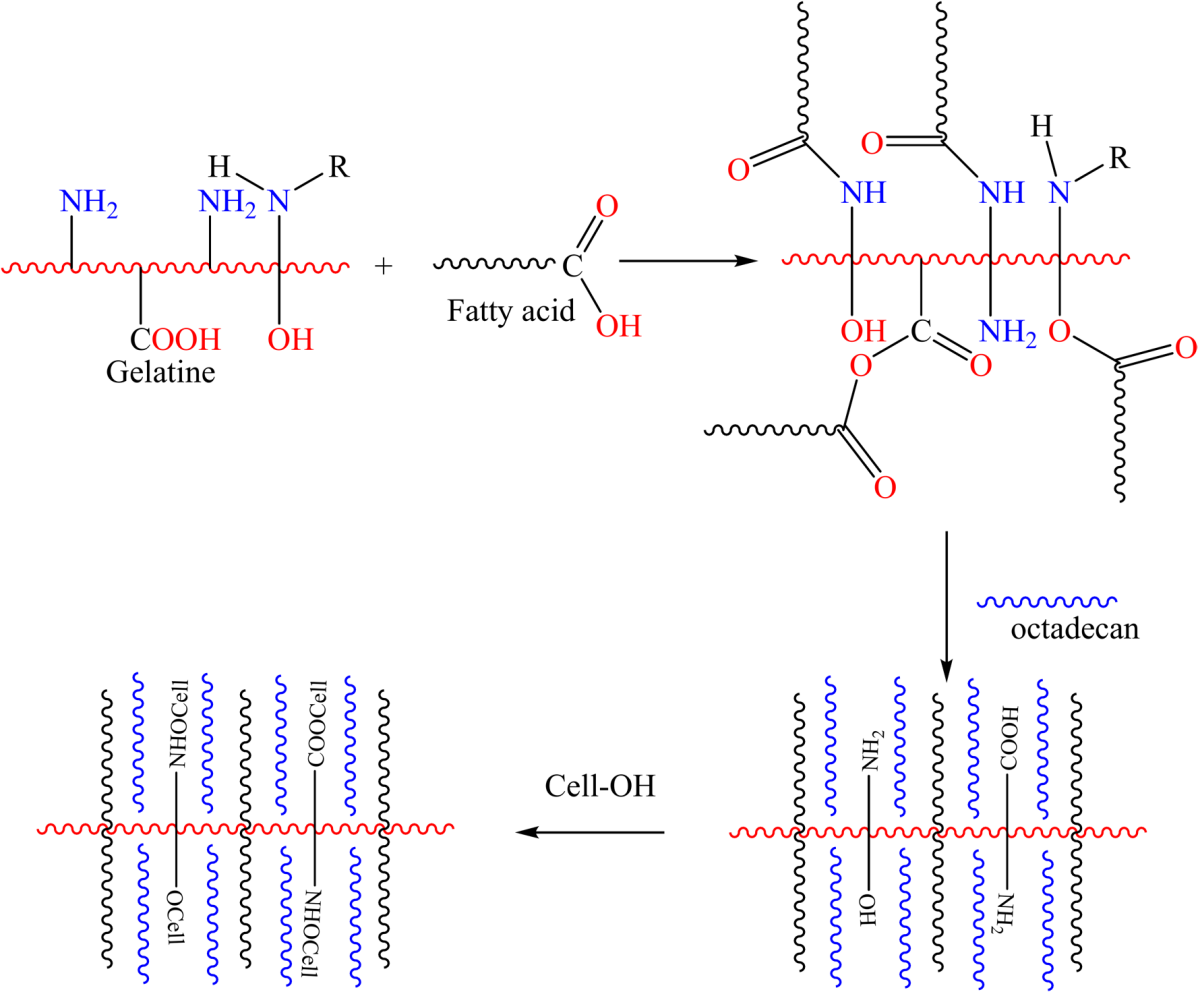
suggested mechanism for the preparation and application of PCM composite.

The treatment solution was prepared with different concentrations (30, 50, and 100 gm/l) of PCM composites and was dispersed in water in the presence of tween 80 (2 g/L) as a surfactant. The solution was homogenized for 10 min at 20,000 rpm using homogenizer. After that the cotton fabric was treated with the PCM solution using the pad-dry-cure method by immersing in the treatment bath at 80 °C for 15 min and then being squeezed with 100% wet pickup. The materials were then dried in an air oven at 100 °C for 5 min. Figure 3 suggested mechanism for the preparation and application of PCM composite.

#### Dyeing of cotton fabric with reactive dye

The dyeing process was done using two methods. a) first method, the fabric was pre-treated with PCM composite and then dyed with reactive dye solution, and b) second method, the fabric was dyed with reactive dye solution and then treated the with PCM composite.

The dyeing procedure was carried out using (3%) reactive dye, 10 g/L NaCl, 5 g/L NaOH at 70 °C for 75 min. then the fabric was dried in oven at 100 °C for 3 min. Finally, the fabric is immersed for 15 min at 50 °C in a soaping bath containing non-ionic detergent.

### Characterization

#### Fourier transform infrared (FT-IR)

FT-IR spectra were recorded on a JASCO FT-IR spectrometer (ATR) was used to analyses the spectrum of the untreated and treated samples. The tester collected transmittance of the infrared in the film between 400 and 4000 cm^−1^ are examined.

#### Scanning electron microscopy (SEM)

Scanning Electron Microscopy HITASHI S-3000 microscope S, at 15-kV acceleration voltage was used to study the fabric surface morphology.

#### Differential scanning calorimetric (DSC)

DSC measurements were carried out on Netzsch DSC 204. 7 mg weighted samples were closed in aluminum pans, and an empty aluminum pan was used as reference. Heating, cooling and heating steps were applied to the pans through the program. Temperature ranged from 0 to 70 °C, with 10 °C/min. The melting temperature and enthalpy ΔH of the peaks recorded during the second heating. Each measurements was repeated 3 time and take the average.

#### Duration index (DI) and total resistance to dry heat transfer

Duration index (DI) (J/cm^3^/K), is a parameter characterizing the material and the temperature at which itis aimed at functioning. Its measure to know that, at a constant temperature during the phase change, how long a PCM will remain^26^.

1 $${\text{DI}} = \frac{{\Delta {\text{H}} \times \rho }}{{\Delta {\text{T}}}}$$

where ΔH is the enthalpy of PCM change of state, ρ is the PCM density and ΔT is the temperature difference between measurable temperature and the temperature of interest (ambient, or body temperature).

The total resistance to dry heat transfer (R) is the insulation value of the clothing systems, and it is related to the textile material on which PCM is applied.

2 $$R = \frac{{\Delta {\text{T}} \times {\text{A}}}}{H}$$

where; A: Area of material, ΔT is the temperature difference between material’s two sides, ΔT = TF−TR (Material’s Front and Rear), and Heat Flow (H). The unit for clothing insulation adopted from studies of hygienic comfort is “clo” (m^2^ °C/W), where 1 clo = 0.155 m^2^ °C/W (zero (0) clo corresponds to a person in a typical business suit and one (1) clo corresponds to a person wearing a naked body)^27–29^.

#### Q-max (warm/cool sensation) and thermal conductivity measurements

Rior to determining the fast dry’s thermal characteristic, each fabric were prepared in compliance with ASTM D1776^30^.

The capacity of heat transport through fabric is referred to as thermal conductivity. Thermal conductivity in this investigation was measured using the KES-F7 standard. The following formula may be used to determine fabric’s thermal conductivity^31^:

$${\text{k }} = \left( {{\text{W }} \times {\text{ D}}} \right)/\left( {{\text{A }} \times \Delta {\text{T}}} \right)$$

where A is the heat plate’s size (25 cm^2^), k is the thermal conductivity (W/cm °C), W is heat flow (W), D is the average thickness of the samples, and ∆T is the temperature differential (heat plate temperature (30 °C)—the cooling base temperature (20 °C) equals 10 °C. The SI unit (W/m-k) may be converted as follows: KSI (W/m-k) = k × 10^2^.

Q-max measurement (feeling hot or cold).

The index that measures how much warmth or coolness a piece of skin feels when it touches cloth is called Q-max. The amount of heat that is lost from the skin to the cloth determines it.

#### Measurement of color strength

A Data color (Data color International 500 reflectance spectrophotometer) was used to determine the color of the textile materials. The Kubelka–Munk equation was used to calculate the color strength (K/S)^32–38^:

$$\text{K}/\text{S }=\frac{{(1-{\varvec{R}})}^{2}}{2{\varvec{R}}}-\frac{{(1-{{\varvec{R}}}_{{\varvec{o}}})}^{2}}{{2{\varvec{R}}}_{{\varvec{o}}}}$$

where R denotes reflectance, K denotes absorption coefficient, and S denotes scattering coefficient.

#### Color fastness properties

The washing fastness properties of colored samples were assessed using the standard ISO 105-C01:2006 test technique^39^. Launderings were done in the Launder-O-Meter (Atlas Electric Co., USA) at 30 °C with soap. The washed specimens were graded on a visual grey scale.

Fatness to rubbing is intended to determine the quantity of color transferred by rubbing from the surface of a colored textile material to another surface. The test is performed using the AATCC crock meter following AATCC Test Method 8-2016^40^. Wet and dry rubbing fastness tests were performed. The test specimens were evaluated using a visual grey scale, and the rubbing fastness rating is graded on a five-point scale^41^. Acidic and alkaline perspiration was carried out according to the test method of AATCC 15-2013 and ISO 105-EO4 (2013)^42,43^. The five fastness ratings could be described as follows: 5 = excellent; 4 = good; 3 = fair; 2 = bad and 1 = extremely poor.

The color fastness measurements to light were determined according to the AATCC Test Method 16-2014^44^.

#### Mechanical properties

According to ASTM Test Method D5035-2011, tensile strength and elongation at a break were measured using a tensile strength apparatus type FMCW 500 (Veb Thuringer Industrie Werk Rauenstein 11/2612 Germany) at 25 °C and 65% relative humidity^45^. The AATCC Test Method 66-2014 was followed in measuring the dry crease recovery angle (CRA)^46^. Using ASTM Test Method D 7127-13, the Surface Roughness measuring equipment SE 1700 was used to test the roughness of the fabric^47^. The cantilever equipment was used to test for stiffness in accordance with ASTM test method D 1388-14e1^48^. The ATSM was used to test air permeability (AP) (D 737-18)^49^. WVP (water vapour permeability) was measured using the ATSM (E96/E96M-16) method^50^.

## Results and discussion

### Optimization and Characterization of synthesized bio-PCM composite

#### Effect of fatty acid concentration

**Fig. 4:**
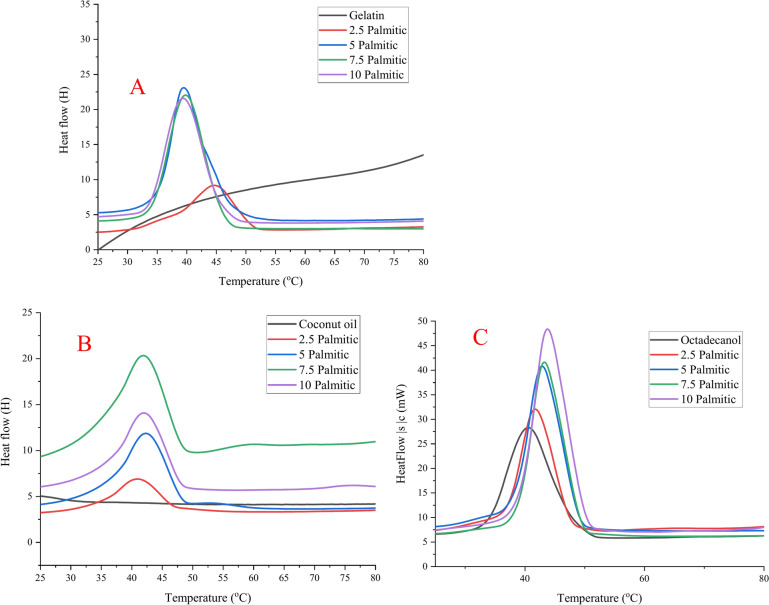
DSC chart for hosting materials using different concentration of palmitic acid with different PCM materials from 2nd heating. (**A**) gelatine and palmitic acid only, (**B**) hosting materials with coconut, and (**C**) hosting materials with octadecanol.

**Fig. 5:**
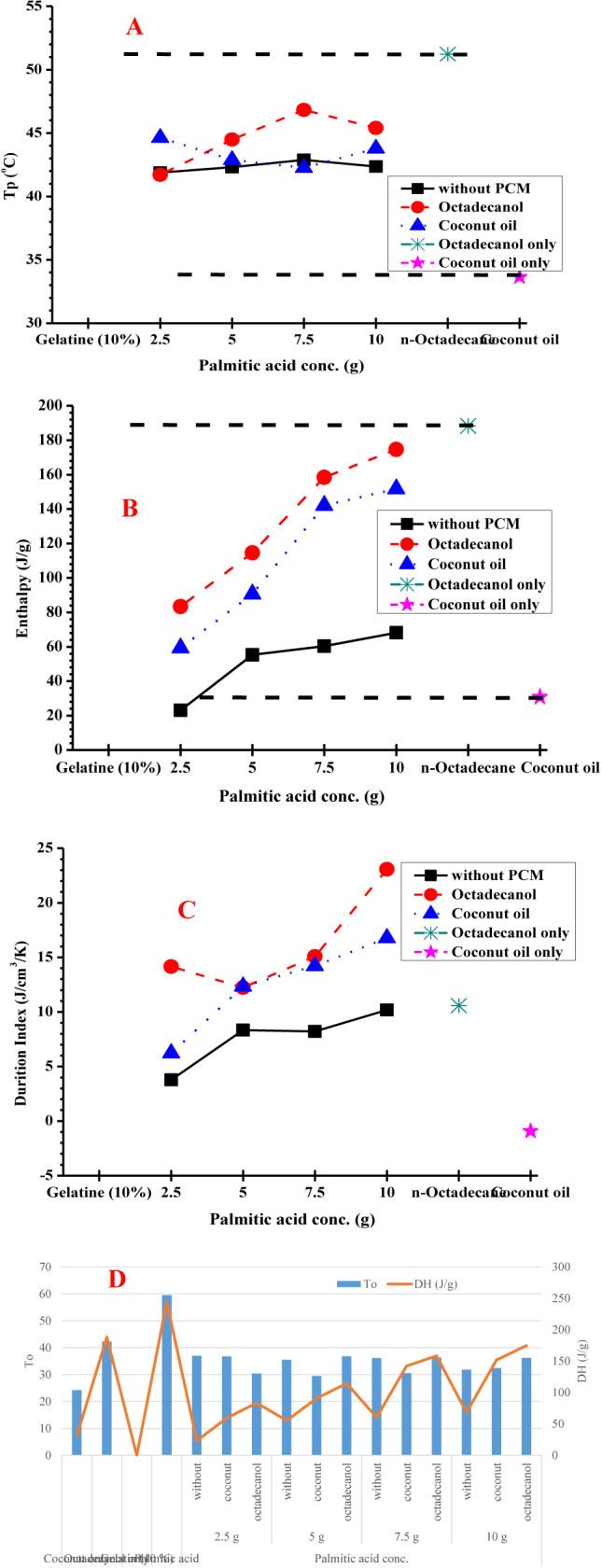
Comparison of DSC recorded data for the palmitic acid at different concentration. (**A**) melting Temperature, (**B**) enthalpy, (**C**) duration index, and (**D**) relation between temperature and enthalpy. Duration Index (DI): based on ΔT from melt point to body temperature (37 °C) and average density of 0.8 g/cm^3^.

During this research the effect of palmitic acid concentration was investigated whether the mixture of fatty acid (palmitic) with gelatine may reach an eutectic (palmitic acid (melting Temp.: 66.9 °C, ΔH: 244.2 J/g)^51^. Octadecanol (melting Temp.: 51.2 °C, ΔH: 188.3 J/g) and coconut oil (melting Temp.: 33.6 °C, ΔH:30.8 J/g). Gelatine were used as 10% in four different ratios (gelatine to palmitic), calculated to the corresponding polymer (gelatine). 2.5, 5, 7.5 and 10 g of palmitic acid to 30 g gelatine (10%). The DSC results from second heating for PCMs are shown in Table 1, Figs. 4 and 5.

**Table 1:** DSC data for hosting materials using different concentration of palmitic acid with/without different PCM materials from 2nd heating.

Palmitic acid conc.	PCM material	2nd heating				
		T_o_	T_p_	T_end_	ΔH (J/g)	DI (J/cm^3^/K)
Palmitic acid conc.						
Coconut only		24.16	33.65	43.82	30.83	− 0.91
Octadecanol only		42.31	51.23	65.32	188.31	10.59
Gelatin (10%)		0	0	0	0	0.00
Palmitic acid		59.47	66.94	84.53	244.19	6.52
2.5 g	Without	36.98	41.89	48.49	23.12	3.78
	Coconut	36.74	44.63	52.82	59.32	6.22
	Octadecanol	30.36	41.71	49.82	83.39	12.23
5 g	Without	35.49	42.31	49.82	55.36	8.34
	Coconut	29.45	42.88	49.18	90.69	12.34
	Octadecanol	36.76	44.49	52.89	114.55	14.6
7.5 g	Without	36.16	42.88	49.35	60.36	8.21
	Coconut	30.59	42.26	46.95	142.1	14.22
	Octadecanol	36.26	46.83	52.36	158.37	15.08
10 g	Without	31.81	42.36	49.64	68.21	10.18
	Coconut	32.38	43.78	50.79	151.73	16.77
	Octadecanol	36.21	45.4	53.13	174.69	23 .08

Hosting material: 2.5, 5, 7.5 and 10 g palmitic acid + 30 ml gelatin (10%). PCM material: 10% coconut or octadecanol.

**T**_**o**_: Onset Temperature, **T**_**p**_: Keeping Temperature, $\Delta$**H**: Enthalpy.

**Duration index**: based on $\Delta$T from melt point to body temperature (37 °C) and average density of 0.8 ${\text{g}/\text{cm}}^{3}$

The first observe from the second heating run that, for PCM-polymer composites prepared from gelatine and palmitic using octadecanol or coconut oil provide increasing in the enthalpy upon increasing the palmitic acid in the host materials. In addition, increasing the palmitic acid concentration up to 10 g results in increasing in the heat storage, so it’s provided good heat storage without further increasing in the palmitic acid concentration in the hosting materials.

In Fig. 5, the PCM-polymer composites are ordered according to the Duration Index. The estimates are based on insulation at 37 °C, which is the temperature of the body. The values of the Duration Index of the hosting materials including both octadecanol and coconut oil are 16.7 J/cm^3^/K and 27.8 J/cm^3^/K palmitic acid for coconut oil and octadecanol, respectively. These materials provide the best material (having the highest Duration Index).

#### FT-IR analysis

**Fig. 6:**
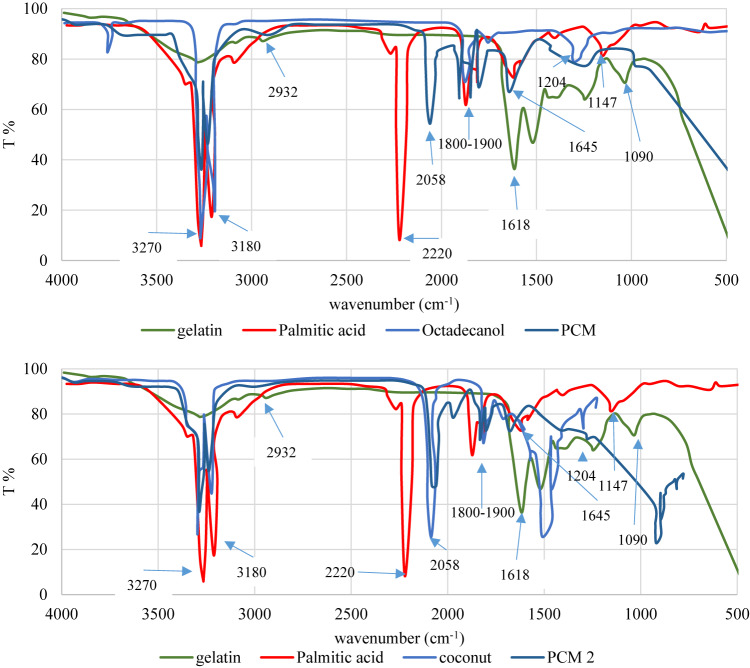
FT-IR spectra for prepared PCM composite with octadecanol and coconut oil.

Utilizing FT-IR, gelatin palmitate was characterized. Figure 6 shows the IR spectra of gelatin, palmitic acid, coconut oil and produced PCM composites.

The results of FT-IR depicted in Fig. 6 showed that the absorption peaks observed at 3270 cm^−1^ for gelatin and 3182 cm^−1^ for the new gelatin derivatives correspond to the intermolecular hydrogen-bonded –OH stretching and –NH stretching in secondary amides (gelatin amide A).

The FTIR spectrum of the Gelatin provide band at 1328 cm^−1^, which is attributed predominantly to the so-called wagging vibration of proline side chains. The 1328 cm^−1^ band in gelatin does not simply represent the carboxyl group, but it is one of a number of bands in the range of 1400–1260 cm^−1^ which are attributed to the presence of type-I gelatin.

gelatin FT-IR spectra exhibit peaks at 2932 cm^−1^, which are associated with –CH stretching peaks. Additionally, another peak was identified as a CH_2_ peak at 1357 cm^−1^. Additionally, Fig. 6 supported the addition of a palmitic acid to gelatin for chemical modification. Following are some examples of how FT-IR spectra reveal chemical alteration for gelatin: O–H: 3270 cm^−1^, C–H: 2932 cm^−1^, C=O: 1747 cm^−1^ (methyl ester), C=O: 1694 cm^−1^ (fatty acid ester), COO: 1618 cm^−1^ and COO: 1433 cm^−1^, and C–O: 1204 and 1147 cm^−1^.

Among edible fats and oils, coconut oil has unique IR spectrum. Figure 6 revealed FTIR spectra of coconut oil confirm that there is no peak at region near 3008 and 1645 cm^−1^. Peaks at these regions correspond to unsaturated double bond (=CH; cis) and –C=C–(cis), respectively. These peaks can be used as an indicative for the unsaturation degree of triglyceride. Coconut oil contained high level of lauric acid (about 50%) and very low level of unsaturated FA of oleic and linoleic acids, therefore, it is not surprising if coconut oil has no peak at region near 3008 and 1645 cm^−1^. In addition, at region ranges of 1120–1090 cm^−1^, due to C–O ester linkage vibration, coconut oil has one peak^52^.

#### Effect of fatty acid type

**Fig. 7:**
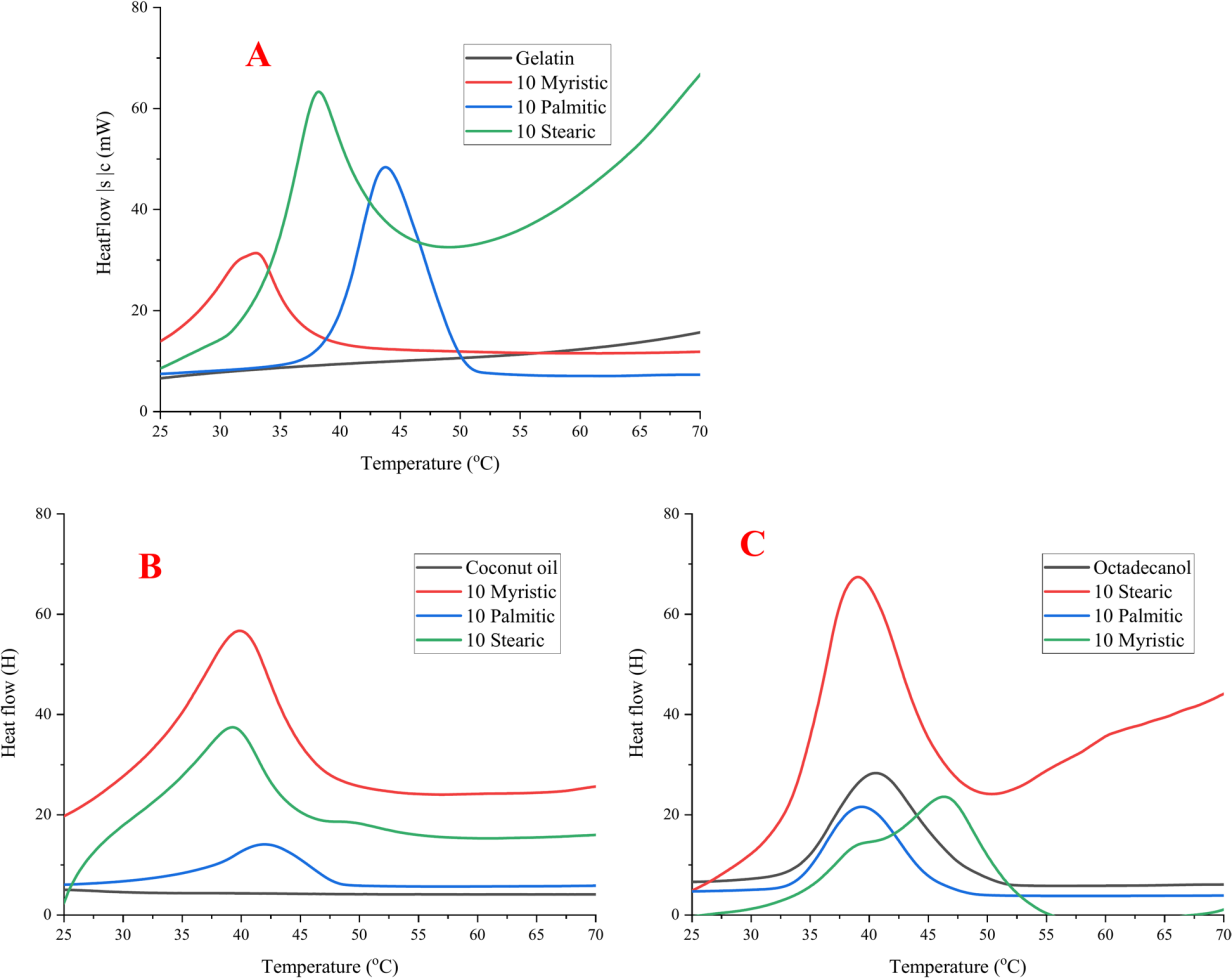
DSC chart for hosting materials using different fatty acids with different PCM materials from 2nd heating. (**A**) gelatine and fatty acids only, (**B**) hosting materials with coconut, and (**C**) hosting materials with octadecanol.

**Fig. 8:**
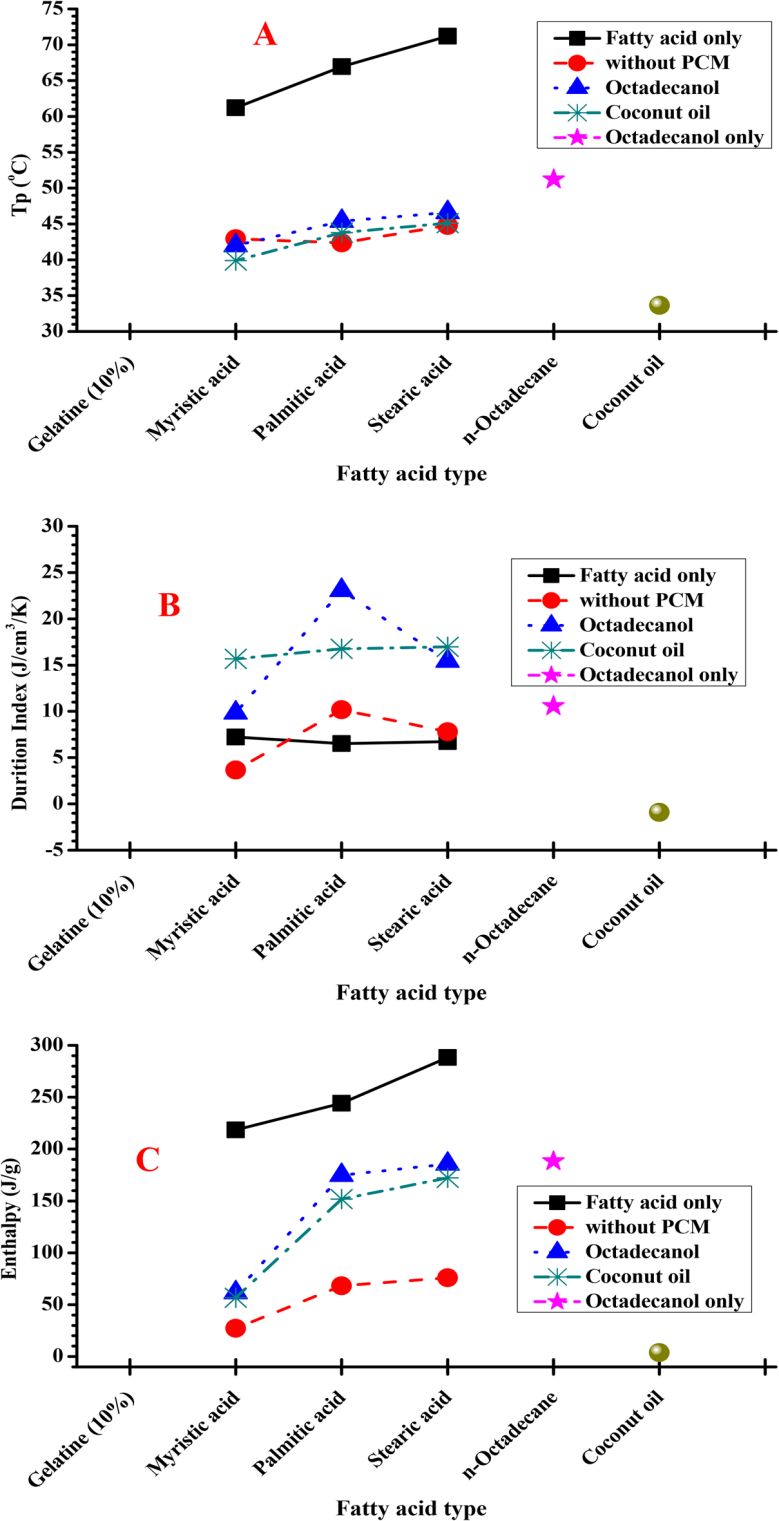
Comparison of DSC recorded data for different fatty acid. (**a**) melting Temperature, (**b**) enthalpy and (**c**) duration index. Duration Index (DI): based on ΔT from melt point to body temperature (37 °C) and average density of 0.8 g/cm^3^.

The DSC results for naturally synthesized composite materials made from gelatine and different fatty acid (myristic, palmitic and stearic acid) in presence and absence of Octadecanol and coconut oil as PCM materials are shown in Table 2 and Figs. 7 and 8. Table 2 clearly shows that the latent heat of the hosting materials increases when the fatty acid backbone increases in the completed composite form. When the biopolymer interacted with fatty acid, the melting point likewise decreased, and it was increased again once Octadecanol was introduced to PCM composite. Phase compliance with the final melting temperature. The best result was obtained by using stearic acid because it has the largest number of carbon atoms in its backbone which caused highly melting point and highly heat storage ability. The results conclude that, the best result was stearic acid because it is the biggest in the number of carbon atoms or not.

**Table 2:** DSC data for hosting materials using different type of fatty acid with/without different PCM materials from 2nd heating.

Fatty acid	PCM material	2nd heating				
		T_o_	T_p_	T_end_	ΔH (J/g)	DI (J/cm^3^/K)
Coconut only		24.16	33.65	43.82	30.8	− 0.91
Octadecanol only		42.31	51.23	65.32	188.31	10.59
Gelatin (10%)		0	0	0	0	0.00
Myristic	My. Only	45.95	61.21	70.21	218.39	7.22
	Without	23.61	42.94	49.36	27.22	3.67
	Coconut	29.14	39.89	49.64	56.65	15.68
	Octadecanol	32.61	41.99	50.79	61.36	9.84
Palmitic	Pa. Only	58.9	66.94	76.82	244.19	6.52
	Without	31.81	42.36	49.64	68.21	10.18
	Coconut	32.38	43.78	50.79	151.7	16.77
	Octadecanol	36.21	45.4	53.13	174.69	15.4
Stearic	St. only	59.47	71.21	84.53	288.39	6.74
	Without	36.21	44.8	51.21	76.01	7.80
	Coconut	36.64	45.11	51.96	172.18	16.98
	Octadecanol	36.81	46.62	53.53	185.74	23.8

Hosting material: 10 g fatty acid (myristic, palmitic and stearic) + 30 ml gelatin (10%). PCM material: 10% coconut or octadecanol.

**T**_**o**_: Onset Temperature, **T**_**p**_: Keeping Temperature, $\Delta$**H**: Enthalpy.

**Duration index**: based on $\Delta$T from melt point to body temperature (37 °C) and average density of 0.8 ${\text{g}/\text{cm}}^{3}$

The DI values of the composite made from both octadecanol and coconut oil suggest that it may be structured to shield the body from the environment’s changing temperature, according to the data in Table 2.

#### Effect of coconut and octadecanol concentration

**Fig. 9:**
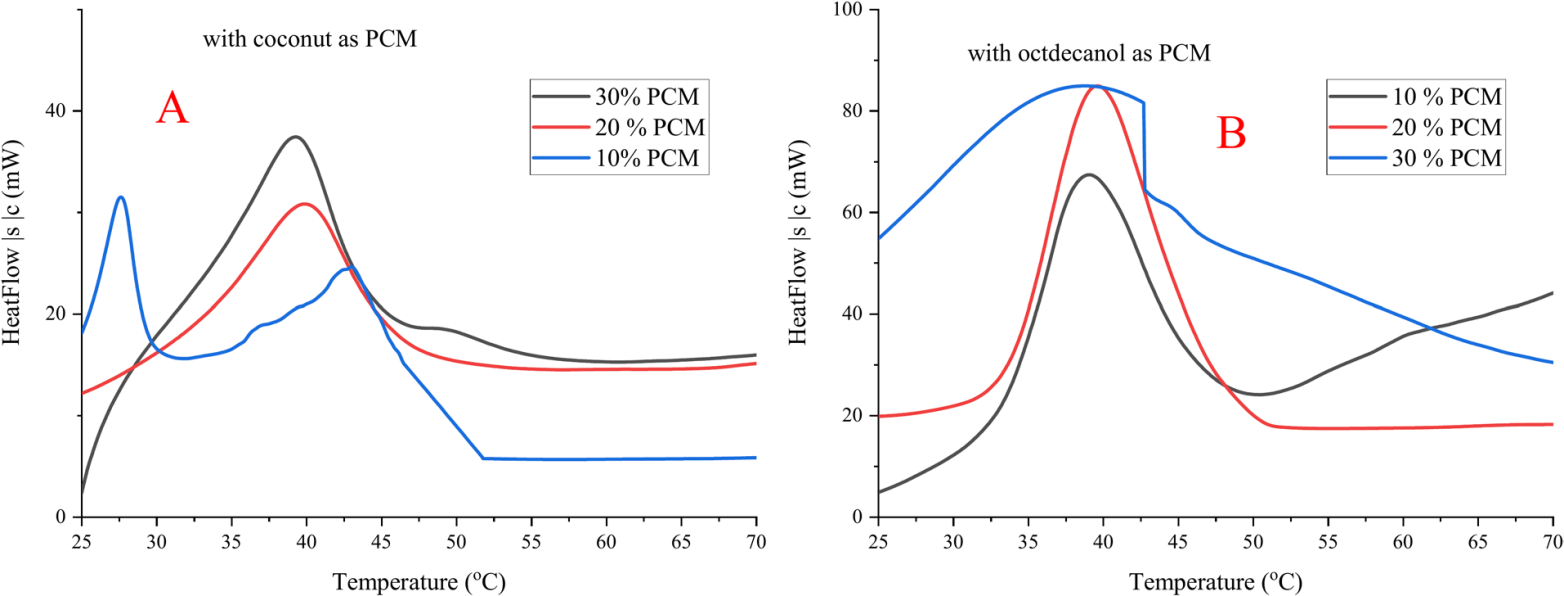
DSC chart for hosting materials using stearic acid with different PCM (coconut (**A**) and octadecanol (**B**)) concentration from 2nd heating.

**Fig. 10:**
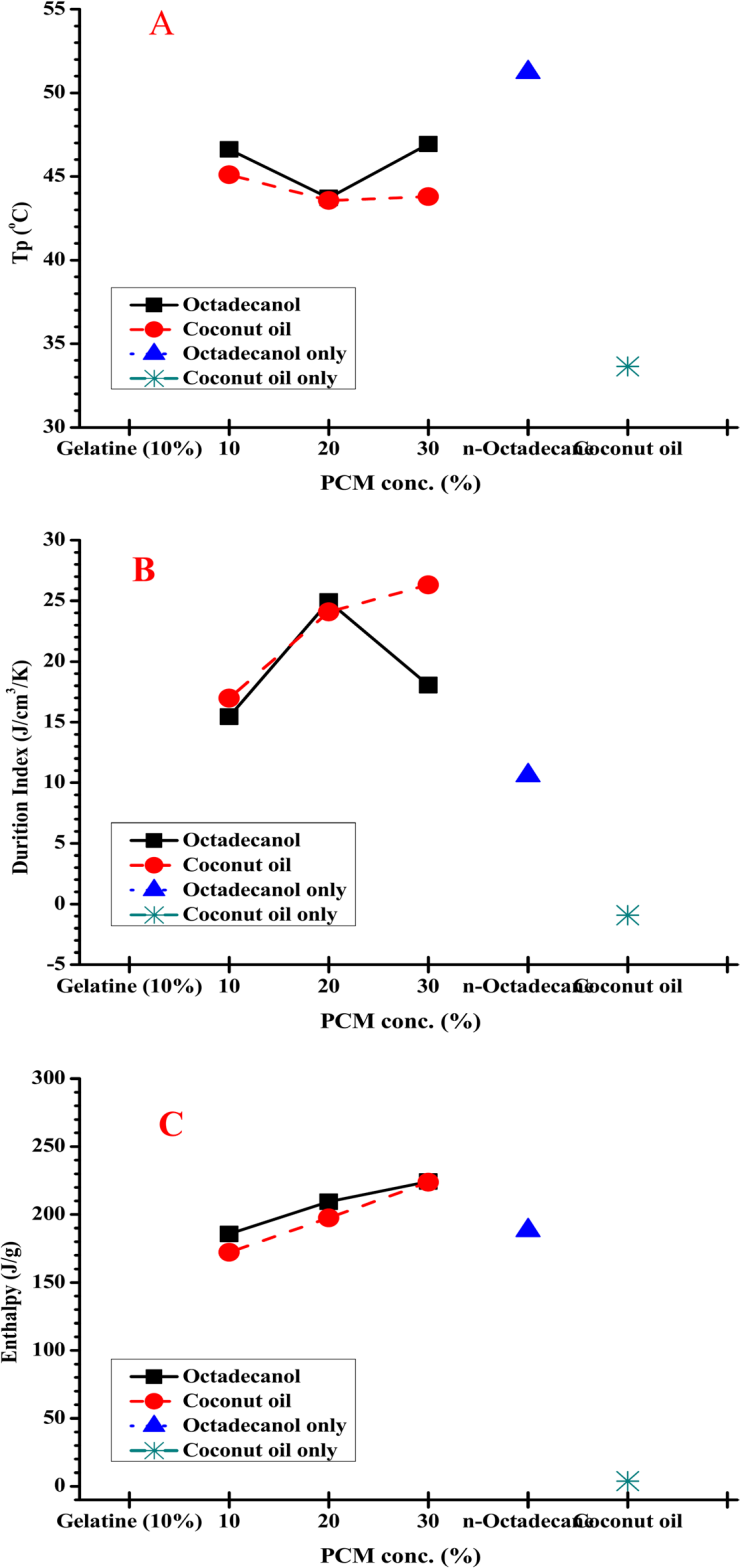
Comparison of DSC recorded data for steric acid with different PCM concentration. (**A**) melting Temperature, (**B**) enthalpy and (**C**) duration index. Duration Index (DI): based on ΔT from melt point to body temperature (37 °C) and average density of 0.8 g/cm^3^.

The DSC results for naturally synthesized composite materials made from gelatine and stearic acid in presence and absence of Octadecanol and coconut oil as PCM materials with different concentration (10, 20 and 30%) are shown in Table 3, Figs. 9 and 10. Table 3 clearly shows that the latent heat of the hosting materials increases when the concentration of PCM material increased in the completed composite form. Figure 9 shows the DSC curve for hosting materials utilising stearic acid at various PCM (coconut and octadecanol) concentrations from the second heating.

**Table 3:** DSC data for hosting materials using different concentration of PCM materials from 2nd heating.

PCM material	2nd heating				
	T_o_	T_p_	T_end_	ΔH (J/g)	DI (J/cm^3^/K)
Coconut only	24.16	33.65	43.82	3.827	− 0.91
Octadecanol only	42.31	51.23	65.32	188.31	10.59
Gelatin (10%)	0	0	0	0	0.00
St. only	59.47	71.21	84.53	288.39	6.74
Without	31.81	45.11	51.21	102.18	10.08
10%					
Coconut	36.64	43.11	51.96	172.18	16.98
Octadecanol	36.81	46.62	53.53	185.74	15.45
20%					
Coconut	36.74	43.56	48.19	197.52	24.09
Octadecanol	36.91	46.72	52.38	209.54	18.05
30%					
Coconut	36.36	43.8	52.01	223.72	24.95
Octadecanol	36.78	46.94	56.57	224.21	26.32

Hosting material: 10 g stearic acid + 30 ml gelatin (10%). PCM material: 10, 20 and 30% coconut or octadecanol.

**T**_**o**_: Onset Temperature, **T**_**p**_: Keeping Temperature, $\Delta$**H**: Enthalpy.

**Duration index**: Based on $\Delta$T from melt point to body temperature (37 °C) and average density of 0.8 ${\text{g}/\text{cm}}^{3}$

The DI values of the composite made from both octadecanol and coconut oil increased with increasing the PCM concentration inside the hosting material suggest that it may be structured to shield the body from the environment’s changing temperature, according to the data in Table 3.

### Characterization of treated fabrics

#### Differential scanning calorimetric (DSC)

**Fig. 11:**
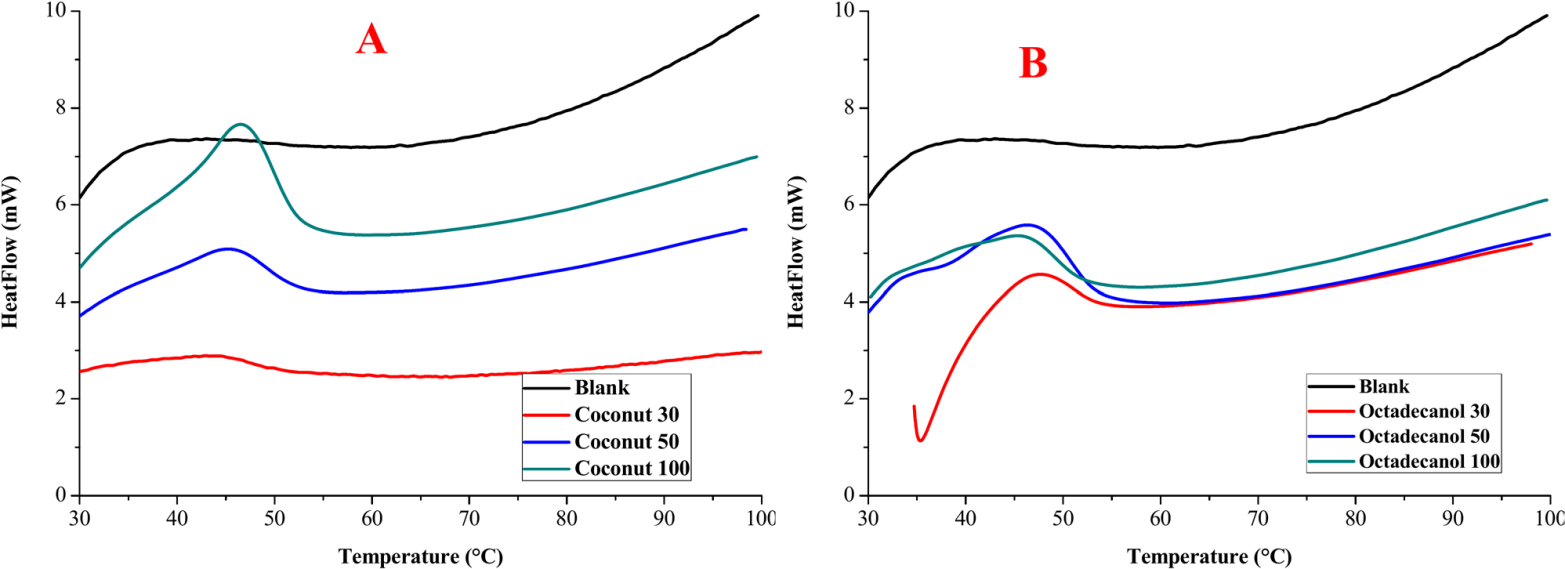
DSC chart for treated fabric using different PCM concentration (in presence of coconut (**A**) or octadecanol (**B**)) from 2nd heating.

Heat is lost to the environment via convection, radiation, and skin evaporation. Table 4 and Fig. 11 displays the DSC findings of cotton fabric treated with gelatine/stearic acid and octadecane or coconut oil. It has been noticed that covered cotton fabric using these composites imparts the thermo-regulating capabilities in contrast to uncovered fabric (Blank). In terms of thermal protection, textiles’ most important job is to maintain a microclimate next to the skin in order to meet any surface’s need for thermoregulatory behaviour^53^. The existing insulating characteristic of the structure can give an increased warm thermal capacity while preserving comfortability when textile apparel is paired with PCM material.

**Table 4:** DSC, Duration index, and Total Resistance results of covered cotton fabric with PCM composite material (Gelatine/Stearic with coconut oil or Octadecanol composite).

Sample description	Thickness (cm)	T_o_ (°C)	T_p_ (°C)	ΔH (J/g)	DI * (J/cm^3^ K)	R ** (clo)	Q_max_	Thermal conductivity
Blank	0.050	34.74	34.86	0.88	0.31	0.406	0.136	0.058
Octadecanol								
30	0.056	33.50	36.19	37.10	10.30	0.208	0.136	0.060
50	0.053	33.96	36.52	50.62	13.34	0.017	0.136	0.057
100	0.062	34.12	37.51	73.32	16.74	0.011	0.136	0.062
Coconut								
30	0.058	34.68	37.95	40.24	15.78	0.206	0.127	0.059
50	0.056	34.96	37.93	54.14	18.68	0.015	0.135	0.057
100	0.067	35.18	41.04	79.60	34.94	0.007	0.119	0.060

With order to increase the thermal insulator qualities and minimise temperature fluctuations, the cotton fabric was wrapped in PCM composite material. Using PCM materials is an efficient way to store and release heat while modifying the ambient temperature^10,54–56^.

Gelatine/Stearic with coconut oil gives more latent heat than coated fabric with Gelatine/Stearic with octadecanol and uncoated fabric. According to estimates of the total resistance to dry heat transfer, cotton fabric coated with Gelatine/Stearic acid/Octadecane or coconut oil composite are more pleasant than those that are untreated. Finally, it can be said that PCM composites may be used to cover cotton fabric, and that the covered fabric outperforms their uncovered counterparts in terms of performance.

#### Dyeing performance

Effect of treatment on the dyeing performance and the vice versa was investigated during this research. Treated cotton fabric using gelatine/stearic/ octadecanol or coconut oil was dyed and investigated for their thermal storage performance and thermal conductivity. In addition, cotton fabric was dyed at optimum condition then treated using gelatine/stearic/octadecanol or coconut oil and investigated for their thermal storage performance and thermal conductivity.

**Fig. 12:**
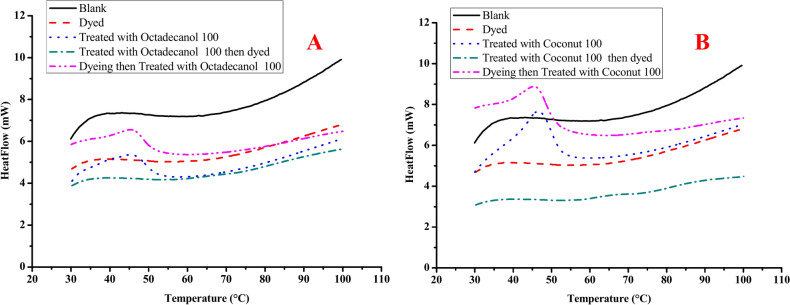
DSC chart for treated fabric using different PCM before (**A**) and after (**B**) dyeing.

Table 5 and Fig. 12 shows thermal storage performance and thermal conductivity for treated fabric (T), treated then dyed fabric (TD) and dyed then treated fabric (DT). It is clear from the data in Table 5 that, dyed fabric then treated using each PCM materials (octadecanol or coconut oil) provide better thermal storage performance and thermal conductivity than treated then dyed fabric. it is may be attributed to that, after dyeing all the functional group on the surface of cotton fabric was reacted with dye molecules, then upon treatment, PCM composite material make a thin film on the surface of dyed cotton fabric which allow the PCM materials to react and produce the effect of thermoregulation properties. From the other point of view, dyeing treated fabric allow the dye molecules to react with hosting materials (gelatine / stearic acid) and free functional group on the surface of cotton fabric and as the dyeing process occurred at 70 °C which convert the PCM from solid phase to liquid phase and make them laying out from the surface led to decreasing the thermoregulated performance^10,57^.

**Table 5:** DSC, Duration index, and Total Resistance results of treated and dyed cotton fabric.

Sample description	Thickness (cm)	T_o_ (°C)	T_p_ (°C)	ΔH (J/g)	DI * (J/cm^3^ K)	R ** (clo)	Q_max_	Thermal conductivity
Blank	0.050	34.74	34.86	0.88	0.31	0.406	0.136	0.058
Dyed	0.058	34.12	36.19	18.55	5.15	0.022	0.136	0.060
Octadecanol 100								
T	0.062	34.12	37.51	82.69	20.72	0.011	0.136	0.062
TD	0.067	34.43	35.53	9.72	3.03	0.214	0.136	0.054
DT	0.067	33.81	36.85	73.32	16.74	0.012	0.136	0.059
Coconut 100								
T	0.067	35.18	41.04	89.73	40.85	0.007	0.119	0.060
TD	0.067	35.37	36.93	10.46	5.14	0.166	0.106	0.051
DT	.069	35.24	39.67	79.60	34.94	0.008	0.135	0.055

*T* Treated, *TD* Treated then dyed, *DT* Dyed then treated.

From Table 6, it can be noticed that the dyeing of the treated fabric provides higher color strength properties than dyeing then treated fabric which could be attributed to the formation of the thin film from the PCM composites which activate the surface with functional groups helps the fabrics to absorb more dyes molecules. While dyeing then treated provides almost similar color strength to the blank dyed fabric. In addition, the slightly increasing in the color strength of the fabric dyed then treated could be attributed to the PCM composite which compact the fabric thickness and causing slightly increasing in the color strength.

**Table 6:** Color strength (K/S) and fastness properties of different treated dyed fabrics.

Sample description	K/S	Fastness properties								
		Washing		Rubbing		Perspiration				Light
						Acidic		Alkaline		
		Alt.	St.	Dry	wet	Alt.	St.	Alt.	St.	
Dyed blank	7.62	4	4	4	3–4	4	3–4	4	4	5
Octadecanol 100										
TD	10.44	4	3–4	4	3–4	4	4	4	4	4–5
DT	7.23	4	4	3–4	4	4	4	4	4–5	5
Coconut 100										
TD	9.52	4	4	3–4	3–4	3–4	3–4	4	4	4–5
DT	7.30	4	4	3–4	4	4	4	4	4	5

#### Thermal conductivity measurement

The thermal conductivity of variably cotton fabrics is shown in Table 5. Both treated fabrics (T) with octadecanol or coconut oil has a substantially greater thermal conductivity than treated then dyed (TD) and dyed then treated (DT). The highest thermal conductivity for T is 0.062 and 0.06 for treated fabrics (T) with octadecanol or coconut oil. The thickness of the fabric affects its ability to transfer heat. That influence is clear from the data in Table 5. It was previously reported that heat conductivity decreases with increasing textile thickness^58–60^. As a result of its greater thermal insulation, the thicker fabric has a lower heat conductivity.

The greater the thermal conductivity rating, the better the fabric’s ability to conduct heat. Treated fabric only (T) had the highest thermal conductivity, whilst treated then dyed fabrics (TD) had the lowest.

A higher Q-max value (warmth or coldness) denotes a colder first contact experience. The Q-max values for the examined samples (T, TD and DT) are 0.136, 0.136, and 0.136, for treated fabric with octadecanol respectively, while it was 0.119, 0.106 and 0.135 for treated fabric with coconut oil respectively. As can be seen, treated fabric using coconut oil had lowest Q-max values than treated fabric using octadecanol in all cases.

Additionally, the fabric thickness after treatment with prepared PCM are reduced because the treatment has made a thin layer on the fabric’s surface, lowering the fabric’s thickness. However, all treated fabrics now have higher thermal insulation qualities since prepared materials can retain temperature.

The initial touch feeling provided by Q-max is essential since it causes a cooling sensation when in contact with human skin. In this investigation, it was noticed that treated cotton fabrics had a cooler touch feel and are thus superior than untreated ones for apparel.

#### Durability measurement

**Fig. 13:**
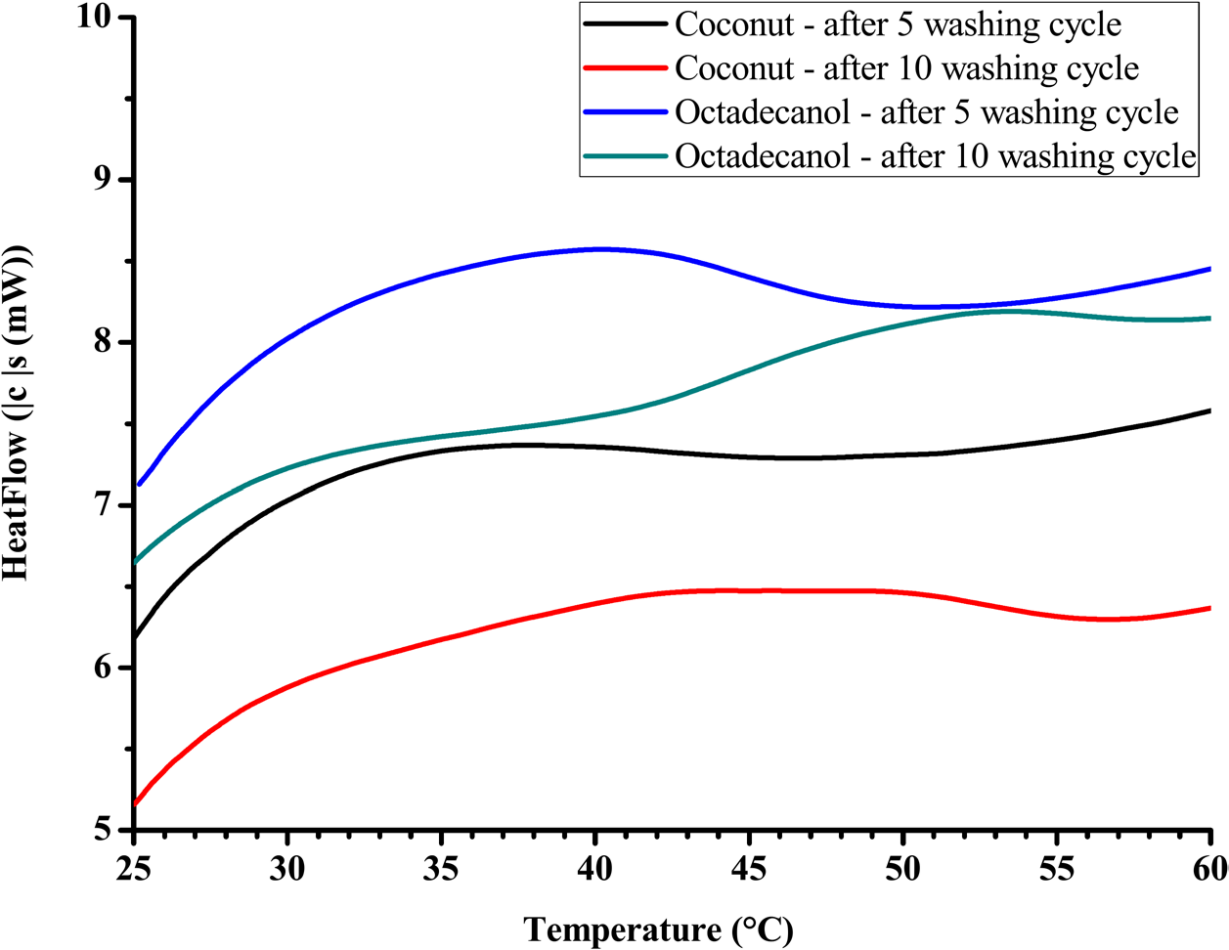
DSC chart for dyed treated fabric using different PCM after different washing cycle.

Table 7 and Fig. 13 show the DSC chart for dyed and treated fabric using different PCM-based composite using (coconut and octadecanol) after different washing cycles (5 and 10 washing cycles). It is clear that the overall characteristics of dyed treated fabric (thermal insulation and color intensity) slightly decreased upon 5 washing cycle and the decreasing was increased upon increasing the washing cycle to 10 cycles. Although this decreasing in fabric characteristics upon different washing cycle, the fabric still has the ability to store the heat and the changing in the color strength still in acceptable value and more than the blank fabric without treatment.

**Table 7:** K/S, DSC, Duration index, and Total Resistance results of treated dyed cotton fabric after different washing cycle.

Sample description	K/S	T_o_ (°C)	T_p_ (°C)	ΔH (J/g)	DI * (J/cm^3^ K)	R ** (clo)	Q_max_	Thermal conductivity
Octadecanol 100								
DT	10.23	34.43	35.53	9.72	3.030	0.214	0.136	0.054
5 WC	9.97	33.98	35.07	6.52	2.657	0.188	0.119	0.047
10 WC	9.60	33.20	34.26	4.37	2.251	0.159	0.101	0.040
Coconut 100								
DT	9.30	35.37	36.93	10.46	5.140	0.166	0.106	0.051
5 WC	8.16	34.91	36.45	7.17	4.508	0.146	0.093	0.045
10 WC	7.91	34.11	35.61	5.85	3.818	0.123	0.079	0.038

*WC* washing cycle.

#### Evaluation of comfortability

Textiles are a material that is frequently used in daily life. On the other hand, surface treatment or multilayer methods are needed for direct outdoor usage of textiles, including natural ones like cotton, for weather protection and waterproofing^61,62^. Due to their innate hydrophilicity and structural instability when exposed to water, these materials are particularly desirable for weathering^63^.

The air permeability of various cotton fabrics has a significant impact on how well they operate. The cloth’s air permeability is significantly influenced by its weight and structure (thickness and porosity).

The void volume in woven textile fabrics is what makes them permeable to air. The air permeability of a textile is determined by the rate at which air moves through a material when there is a pressure differential between the two surfaces of the fabric^64^.

**Fig. 14:**
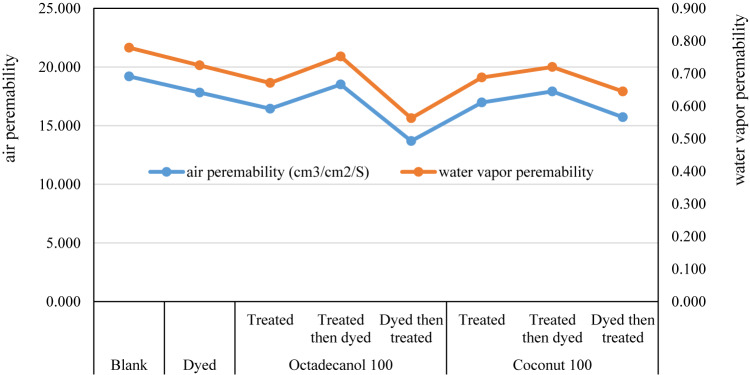
air permeability and water vapor permeability of treated and untreated fabrics.

Figure 14 shows the air and water vapour permeabilities of treated and untreated textiles, and the data sources show that both of the tested parameters slightly dropped after treatment but remained within acceptable ranges. This demonstrates that both treatment and dyeing had no impact on the comfortability of the cloth.

#### Morphological behavior

**Fig. 15:**
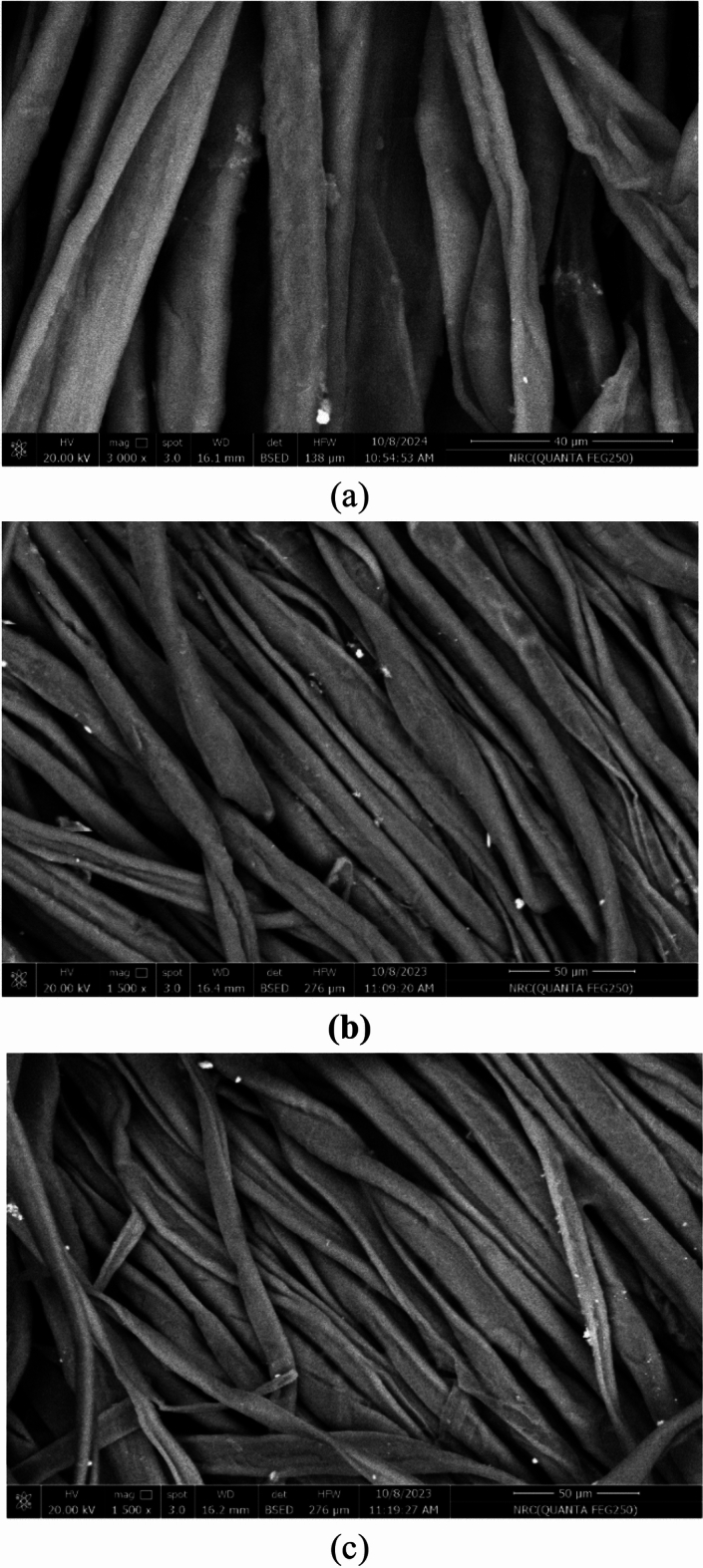
morphological behaviour of cotton textiles. (**a**) untreated fabric, (**b**) treatedS fabric with PCM (Octadecanol 100), (**c**) treated fabric with PCM (coconut oil 100).

The morphological behaviour of cotton textiles was examined after being treated with PCM composite material before dyeing (see Fig. 15). It is clear from that treatment using both composites (using coconut and octadecanol) provide the presence of homogeneous thin film on the surface of the cotton fibers. This thin film causing homogeneity in the filling the gabs in the surface of cotton fiber which responsible for the enhancement of thermal properties as well as the heat storage property.

#### Mechanical and physical performance

The tensile strength, elongation at break, roughness, and crease recovery angle of cotton textiles in the warp and weft directions were examined before and after being treated with PCM composite material before and after dyeing. The Young’s Modulus is an inherent characteristic of all materials, which remains constant but can be influenced by temperature and pressure. It represents the stiffness of a material, indicating how easily it can be bent or stretched. It is defined as the ratio of tensile stress (force per unit area) to tensile strain (elongation at a break). The increase in Young’s Modulus observed after treating the fabric with PCM composite coating resulted in reduced rigidity of the fabric.

Table 8 displays the results, and it shows how differences in process of treated and dyeing fabric lead to changes in the physicomechanical properties of cotton fabric. While only slightly improving in the crease recovery angle and fabric roughness, the treatment dramatically increased the tensile strength and elongation at the break. This shows that the gelatin/stearic polymer material used as the hosting was extensively absorbed into the textile fabrics’ microstructure, resulting in a thin coating layer on the fabric’s surface that was responsible for the observed alterations^65–68^.

**Table 8:** mechanical and physical properties of treated and untreated fabrics.

Sample descriptio	Air permeability (cm^3^/cm^2^/S)	Water vapor permeability	Tensile strength (kgf)	Elongation at a break (%)	Young’s modulus (kgf/mm^2^)	Roughness	CRA
Blank	19.197	0.779	55.50	37.0	34.30	17.17	114
Dyed	17.819	0.725	57.68	41.8	44.43	17.20	121.8
Octadecanol 100							
T	16.442	0.671	60.43	45.3	61.82	17.29	127
TD	18.508	0.752	56.59	39.4	39.37	17.18	117.9
DT	13.687	0.563	67.98	51.0	69.54	19.45	142.9
Coconut 100							
T	16.973	0.688	66.84	43.7	35.68	17.27	127
TD	17.915	0.720	62.12	41.9	58.96	17.28	118.5
DT	15.718	0.645	75.19	49.1	40.13	19.42	142.9

*T* Treated, *TD* Treated then dyed, *DT* Dyed then treated.

The covalent chemical connection, which also led to the creation of an intensive network with a high degree of cross-linking, was most likely the root cause of the significant increase in the crease recovery angle.

## Conclusion

Smart fabrics have gained popularity in recent years, with phase change materials (PCMs) being incorporated into textile structures to create thermoregulated fabrics. PCMs are thermal energy storage materials that can store and release large amounts of latent heat during phase change. To address melting and leaking issues, hosting materials have been developed to retain PCMs within their network. Chemically bonded hosting materials are preferred as they make the surface permanently treated. PCMs used in textiles are typically microencapsulated into a polymer to avoid material leakage. To address this issue, natural polymers like gelatin have been investigated as an alternative shell. Gelatin is a non-toxic and biodegradable polypeptide with a high molecular weight, making it a promising alternative to traditional PCMs. This study aims to investigate the use of organic coconut oil as a natural PCM for textile applications as a low-cost, sustainable alternative to current PCMs, comparing it with recognized PCMs like octadecanol.

This research investigates the mechanical and physical performance of cotton fabrics treated with gelatine/stearic acid, octadecane or coconut oil. The results show that covered cotton fabric using these composites imparts thermo-regulating capabilities in contrast to uncovered fabric. Textile apparel is crucial for maintaining a microclimate next to the skin, and the insulating characteristics of the structure can provide increased warm thermal capacity while preserving comfortability when paired with PCM material.

To increase thermal insulator qualities and minimize temperature fluctuations, cotton fabric was wrapped in PCM composite material. Gelatine/Stearic with coconut oil gives more latent heat than coated fabric with Gelatine/Stearic with octadecanol and uncoated fabric. Cotton fabric coated with Gelatine/Stearic acid/Octadecane or coconut oil composite are more pleasant than those that are untreated.

The dyeing performance of treated cotton fabric was investigated for thermal storage performance and thermal conductivity. The results showed that treated fabric then treated using each PCM materials (octadecanol or coconut oil) provided better thermal storage performance and thermal conductivity than treated then dyed fabric. The treated fabric also provided higher color strength properties than the untreated fabric.

The thermal conductivity of variably cotton fabrics showed that both treated fabrics with octadecanol or coconut oil had substantially greater thermal conductivity than treated then dyed and dyed then treated fabrics. The treated fabric only had the highest thermal conductivity, while treated then dyed fabrics had the lowest.

Durability measurements showed that the overall characteristics of dyed treated fabric slightly decreased upon 5 washing cycles and increased upon increasing the washing cycle to 10 cycles. However, the fabric still has the ability to store heat and the changing in color strength remains acceptable value.

Morphological behavior of cotton textiles after being treated with PCM composite material before dyeing revealed that treatment using both composites (using coconut and octadecanol) provides the presence of a homogeneous thin film on the surface of the cotton fibers, which enhances thermal properties and heat storage.

## Data Availability

The datasets generated and/or analysed during the current study are available in the the manuscript.
